# Satisfaction with in-patient child and adolescent psychiatric treatment: development and psychometric properties of the BEST questionnaires for adolescents and for parents

**DOI:** 10.1186/s13034-021-00395-1

**Published:** 2021-09-04

**Authors:** Ferdinand Keller, Alexander Naumann, Jörg M. Fegert

**Affiliations:** 1 Department of Child and Adolescent Psychiatry and Psychotherapy, Ulm University Hospital, Steinhövelstr. 5, 89075 Ulm, Germany; 2 Fachklinik Kinder- und Jugendpsychiatrie, MVZ Wichernstift, Spadener Weg 5, 27607 Geestland, Germany

**Keywords:** Patient satisfaction, Parent satisfaction, Child and adolescent psychiatry, Scale development, Exploratory factor analysis, Bifactor model, Children’s rights, Treatment participation

## Abstract

**Background:**

Interest in the assessment of patient satisfaction with in-patient psychiatric treatment has steadily increased and several measurement tools are available for the quantification of patients’ experience. However, they are often uni-dimensional or focus mainly on therapeutic relationship and environment, and neglect other important issues such as information about treatment and participation. The BEST questionnaires were developed as comprehensive instruments that include items on all of the mentioned topics. The present study evaluates the psychometric properties of the BEST in a version for adolescents and for parents. Furthermore, the dimensionality of the satisfaction ratings is analyzed.

**Method:**

Descriptive statistics were applied to data of 1582 adolescents (mean age = 15.0 years, SD = 1.65; 62.4% female) and 1998 parents/guardians assessed in seven in-patient units across Germany. The factorial structure of the BEST questionnaires was determined by exploratory and confirmatory factor analyses, including a bifactor model.

**Results:**

The psychometric quality of the scales was strong. Correlations with another assessment instrument of patient satisfaction were good to high, indicating good convergent validity. Exploratory factor analyses revealed three factors in adolescents that were labelled as: Therapeutic relationship, environment, and general satisfaction and treatment success. For parents, the same three factors could be distinguished. Confirmatory bifactor models suggested that the vast majority of variance was accounted for by the general factor; the three specific factors provided some additional information. Agreement between the subscales of adolescents and their parents was only moderate. Parents were usually more satisfied.

**Conclusions:**

The BEST questionnaires can be considered as reliable and valid instruments to not only assess the “classical” aspects of patient satisfaction, but to also assess newer fundamental topics such as children’s rights and treatment participation. For scientific usage, the total score seems superior because of the high explained variance by the general factor, but the subscale scores provide further information. The use of single items seems advantageous for quality management purposes.

## Introduction

Consumer satisfaction is a central issue in the evaluation of services and has become a key indicator of the quality of health care [1, 2]. The assessment of patient satisfaction is part of quality certification systems such as the KTQ in Germany, and is used to benchmark services in some countries, e.g., in the U.K., [3], and in the U.S. Part of the Patient Protection and Affordable Care Act included rules that incentivize hospitals to improve patient satisfaction by offering increased reimbursements [4]. In the case of serious incidents (harassment of hospital staff and sometimes even direct injuries of hospital personnel) following patient dissatisfaction, the identification of important determinants of dissatisfaction can be a major concern for the health policy [5]. Therefore, there is growing interest within health care providers and regulators about having access to quantitative information regarding the quality of health care services [1].

However, rising interest and the occasional pressure are not only seen within the administrative and policy areas. The second reason for the crucial role of patient satisfaction is related to the consequences for the treatment process. Treatment satisfaction is considered to have positive effects on the treatment adherence, i.e. keeping the child and the family engaged in treatment, which is associated with treatment completion and thus promotes the chance for a positive outcome [3, 6]. It also has been argued that satisfaction ratings may serve as a direct feedback for the therapist and may help to enhance the quality of the interventions [7]. Additionally, it is an agreed endpoint in outcome research [2].

While there appears to be large agreement on the usefulness of capturing the feedback of patients, there is still “a long way between collecting feedback and putting it to good use” [3, p. 435] and an unvalidated ad hoc measure cannot stand on face validity alone [8]. The intended “good use” requires a reliable and valid method to measure satisfaction. It should cover different, practical and relevant domains of satisfaction, and good measurement is a prerequisite of an appropriate quantified interpretation [3, 6, 9]. In an early review on the findings of patient satisfaction research, Lebow [10] summarized that the reliability of satisfaction reports has only been assessed in a few studies and that they appear reliable, but that reliability is likely to be a problem if scales are short. Furthermore, the lack of theoretical foundation for instruments assessing patient satisfaction has been criticized repeatedly [2, 11]. The major patient satisfaction theories were published in the 1980s and still the construct has little standardization [2]. There have been attempts to develop theoretical explanations for how satisfaction ratings may emerge, e.g., Linder-Pelz ([12]; for an overview on theories see Gill and White [2]), but this theoretical background was seemingly not used in constructing assessment instruments except in some newer instruments, e.g., an expectations questionnaire developed by Bowlings et al. [13]. Further progress in theories regarding what patients expect from (medical) treatment has been provided by research on the effectiveness of placebos [14], but to our knowledge, such theories had no direct influence on satisfaction research in the mental health area. Assessment instruments have been developed mostly on the background of sound practical experience, clinical needs and interests, and on an expert basis for child and adolescent mental health treatment [9, 15–18]. Some studies used existing instruments such as the Client Satisfaction Questionnaire (CSQ-8 [19]), an unidimensional scale that was already established in a variety of treatment settings in health and mental health services [8, 9, 20].

The following points are two major problems that are commonly discussed in connection with the development of suitable assessment instruments. The first issue is concerned with which domains of patient satisfaction should (and can) be covered by the instrument? Sawyer et al. [1] developed a conceptual framework for measuring the quality of health care delivered to adolescents in hospitals. The authors identified eight domains extracted from 22 studies and finally suggested a set of 14 indicators (half for adolescents, half for parents) that set the stage to develop measures to populate these indicators, as a next step. A multidimensional hierarchical scale for measuring health service quality was developed and empirically tested by Dagger et al. [21]. In mental health care research, models are less complex and domains of satisfaction have been extracted mostly by empirical approaches [principal component analysis (PCA) and factor analyses (FA)]. Several studies found their questionnaire to be unidimensional [22, 23], but most studies report the existence of two or three empirically distinguishable factors: “relationship with therapist” and “benefits of therapy” [6] or the factors “relationship”, “privacy” and “session activity” in the Child and adolescent service experience (ChASE) questionnaire [15]. The multidimensional instrument developed by Garland et al. [9] identified four factors: Counsellor qualities, meeting needs, effectiveness, and counsellor conflict. The latter factor, however, seems to be a negative version of the first factor, and both seem to reflect a therapeutic relationship in a more psychotherapeutic context. The Therapy Evaluation Questionnaire (TEQ [18]) consists of three subscales for the patient version labelled “treatment success”, “relationship with therapist”, and “general treatment conditions”.

The “Experience of Service Questionnaire” (ESQ) was used countrywide in the U.K and was found to comprise the factors “satisfaction with care” and “satisfaction with environment” [3]. In a critical review of research literature on child and adolescent satisfaction with psychiatric care, Biering [24] identified three universal components: “satisfaction with the environment and the organisation of the services”, “satisfaction with the adolescent–caregiver relationship”, and “treatment outcome”.

Some limitations of simple factor analyses are evident. First, specific factors can only be extracted if the relevant items addressing them are included in the questionnaire. Instruments that aim to evaluate the treatment experience of patients often focus on interaction with therapists and staff, but neglect other aspects of quality such as the satisfaction with the environment. Secondly, questionnaires are to be short enough to be filled-in quickly by the respondents. Thus, even if one or two items may assess a domain such as hotel quality, no proper factor solution for this domain can emerge. Even if there is any sort of expectation of some multi-dimensionality in a short (and broadly constructed) scale, some authors prefer to have a single overall satisfaction score, e.g., [22, 23, 25]. A third point of criticism addresses the conceptual structure of the construct satisfaction. The two or three components that have been distinguished in empirical research with (mainly) PCA could often be labelled adequately, but the correlations between these components were usually high. Several studies discussed a generally positive or negative tendency in answering the satisfaction items [9, 10, 26]. Similarly, Brown et al. [3] considered the responses of the satisfaction items to be influenced by a strong ‘halo’ effect that represents the overall positive or negative affect towards the treatment one has received. Testing these assumptions within a factor model would need a second-order factor model or the inclusion of a method factor. Another straightforward method to help differentiate between a general factor and remaining specific factors is the use of bifactor modelling [27, 28]. This approach allows for new insights into the factorial structure of highly correlated dimensions and has been applied in several research areas, e.g., in the potential distinction between the two closely related cognitive and somatic symptom dimensions in the assessment of depression [29, 30]. Although it is straightforward, these factor models have not yet been applied in satisfaction research and will be one of the key objectives in this study.

One study analysed written suggestions in free-text format about specific service improvements, that were obtained from children and adolescents in outpatient mental health care. A qualitative content analysis revealed three overarching themes: “accessibility”, “being heard and seen”, and “usefulness of sessions” [31].

The second problem has to do with the empirical observation that item and sum scores are usually moved to the positive side (“ceiling effect”) and there is reason to assume that patient are overly positive in answering the questions. Several explanations have been proposed for this observation. There may be psychological reasons for this. For instance, patients see their stay in a generally positive or negative way, as Williams et al. [26] derived from their qualitative interviews. Garland et al. [9] found some anecdotal evidence for the theory of cognitive dissonance, i.e., in short, that adolescents who remain in treatment on their own choice are likely to report high satisfaction to justify their commitment of time and effort. Brown et al. [3] also speaks about the indications for a strong ‘halo’ effect, i.e. a generally positive or negative affect towards one’s treatment. Independently from the validity of these theoretical explanations, evaluation studies using an instrument that yields mostly high positive scores lack clinical utility because high scores give little information on what should be improved. For statistical analyses, the reduced variation of scores limits the comparison of subgroups who may have differences in satisfaction scores, and the calculation of relevant predictors of patient satisfaction. Therefore, unidimensional scales such as the CSQ-8 (which often yields high positive scores in patient groups) do not allow for more detailed analyses of how different dimensions of satisfaction are related to specific outcomes [9, 20]. A methodologically oriented solution to this problem may be to change the wording of the response categories. Following examples from marketing research, a proposal could be to extend the positive end of the scale by including categories such as “excellent” or “one of the best I ever experienced”. Another approach to overcome this ceiling effect was suggested by Längle et al. [32] in a satisfaction questionnaire for adult psychiatric patients. The authors formulated the items in form of a request in the event that they would return to treatment, e.g., if I were to return, the therapist should take me more seriously. This wording of the items was found to reduce ceiling effects and to increase the variance of responses [32].

Our own attempt to develop a questionnaire to assess satisfaction with in-patient stays started around the year 2000 with a review of existing German instruments [33] and an interdisciplinary medico-legal research project. This was funded by the Volkswagen Foundation and comprehensively published in Rothärmel et al. [34], with a main focus on patient information, participation and children’s rights in treatment situation. The above-cited Anglo-Saxon literature from the same time [6, 9, 16, 17] mainly dealt with ambulatory treatment and social work or it was not available and therefore only partially influenced the development of our instrument. Our collection of items was based on: (a) a comprehensive review of published and unpublished nationally used assessment instruments and inspection of international instruments, (b) the pioneering approach of the Marburg group [35], and (c) a qualitative component with focus groups including adolescents and clinical experts. Furthermore, central domains of our research project on children’s rights were missing in established assessment instruments, in particular treatment participation, information about own problem, medication, coercive measures etc., and respect of privacy that have been included in our first form of the “Broad Evaluation of Satisfaction with Treatment” (BEST). The BEST was developed in three versions: for children (BEST-C), for adolescents (BEST-A) and for parents (BEST-P) (for reasons of space and minor conceptual differences, the BEST-C (recommended usage of up to 12 years) is not included in this paper). These issues are important additions to the usual health care perspective where patients are seen primarily as a customer. However, the approach and methodology of the research itself seems to have more similarities than differences between health care and mental health care evaluation studies [36]. In addition, items should be formulated in a way for both adolescents and parents. Finally, our questionnaire should deal with the problem of ceiling effects and the chosen approach was the statement in form of a request (“the food should be better”), following Längle et al. [32]. Beyond this methodological goal, the formulation of the statement as a request also reflects the idea of co-creation and that adolescents have an active part in the treatment process.

This first version of the BEST was used until 2015 and some main results based on large samples of adolescents and parents were published in Keller et al. [37]. The main findings were that the items proved valid in assessing satisfaction, the psychometric properties of the items were good, and the correlation with an established assessment instrument of patient satisfaction was good to high. Exploratory factor analyses revealed five factors in adolescents that were labelled as: Therapeutic relationship, environment, over-all satisfaction, perceived attention and information, and schooling. For parents, three factors could be distinguished: Relationship to therapist, environment, and over-all satisfaction. The agreement between the subscales of adolescents and their parents was only moderate [37].

After extensive practical experience in hospitals, there were requests for shortening the instrument and ideas about some text changes in the item content. Based on our yearly statistical and psychometric analyses of the data for hospital quality reports, a revision of the wording in some items seemed advantageous. The reduced and slightly revised version of the adolescent and of the parent version of the BEST is evaluated in this article.

The aims of the present study were two-fold. The first aim was to examine the psychometric properties of the revised and shortened version of the BEST questionnaire including item evaluation, item-total correlation and factor structure (exploratory factor analyses). Likewise, these new results of the BEST should serve as a replication of the main results of Keller et al. [37] for the “long” versions of the BEST for adolescents and for parents. Concerning validity, the relation to sex and age should be determined and the convergent validity by correlating the BEST with the established Therapy Evaluation Questionnaire (TEQ) [18] questionnaire. In addition, we were interested in the degree of agreement between adolescents and parents’ ratings of satisfaction.

The second aim addressed the dimensionality of the BEST in both versions: is a factor model with different but correlated dimensions favourable, or is the assumption of a common underlying factor more plausible? This was done by advanced testing of the factorial structure with bifactor models and the application of statistical indices to determine how reliable the subscales are after the general factor has been partialled out. In the case of a strong general factor (‘halo effect’), the factor loadings of all items on the general factor should be strong and the explained variance of the general factor should be high; consequently, the reliability of the specific factors (subscales) is expected to be low.

## Methods

### Procedure

Data was collected in seven departments of child and adolescent psychiatry in hospitals of several German states between 2016 and 2017. Questionnaires were provided by the local quality management staff and distributed to the inpatient wards and day clinics of the hospitals. At the end of their stay, participants were asked to fill out the questionnaires. The questionnaires were put in an envelope, closed, and sent in envelopes to Ulm for data management and statistical analysis. All patients and parents were informed about the assessment and gave their written informed consent. The Institutional Review Boards of the University of Ulm approved the study.

For a better understanding of the German system in child and adolescent psychiatry (CAP), it should be added that “there is a broad and well differentiated medical and youth welfare system for treatment and support of children and adolescents with mental disorders in Germany. Roughly 150 specialized units with more than 6300 beds for inpatient/day care treatment are run by hospitals plus 30 University clinics. All costs are covered by health insurance, so services are available for every family or child.” [38]. Overall, the average duration of stay in CAP units in Germany in 2017 was 34.4 days [39]—this number includes short emergency stays (3 days or less). Thus, the majority of participants can be expected to have “lived” in the CAP unit for 1 to 3 months. Treatment in CAP includes individual psychotherapy (mainly cognitive behavior therapy and/or psychodynamic psychotherapy), parent and family related interventions, psychopharmacological treatment, functional therapies (occupational therapy, physiotherapy, orthopedagogy, art therapies), and several trainings [40].

### Participants

In total, 1661 questionnaires from adolescents und 2136 from parents/carers were assessed. Few questionnaires (0–3 per clinic per year) were excluded due to obvious endorsement of patterns, e.g., zig-zag-patterns. Furthermore, some participants did not finish filling out the questionnaire or in some questionnaires, many items were missing. On the other hand, there is a considerable number of items that cannot be answered under some circumstances, e.g., if no medication received, no weekend leave, no family sessions, no school visit. Taking these potential reasons for structural missing data into account, we decided to include all questionnaires with a maximum of missing data in seven items (out of 27) for adolescents and in six items (out of 22) for parents. Thus, the final sample size for adolescents was *n* = 1582 (95.2% of the full sample) and *n* = 1998 for parents (93.5% of the full sample). For the calculation of the correlation coefficients between adolescent and parent subscales, there were *n* = 815 paired questionnaires available. The average age of the adolescents was 15.0 years (*SD* = 1.65) and there were 37.6% boys and 62.4% girls. For the parent sample, the mean age of their child was 13.2 years (*SD* = 2.98) with an about equal ratio of boys and girls (50.4% female).

To examine the convergent validity of the BEST, the TEQ [18] was assessed along with the BEST in a hospital in Northern Germany (not included in the sample described above). In total, 88 questionnaires for adolescents and 90 for parents were available. After applying missing data criteria (same as above for BEST and a minimum of 50% of items endorsed in the TEQ, after [18]), the correlation coefficients between BEST and TEQ were based on *n* = 80 for adolescents and *n* = 82 for parents. The average age of the adolescents was 15.4 years (*SD* = 1.33) and 69.6% were female.

### Assessment instruments

The BEST for adolescents consists of 27 items that are answered on a scale ranging from 1 = “strongly disagree” to 5 = “strongly agree”. Additional categories are given in some questions, e.g., “not on medication”. The initial three items and items 26 and 27 are asked (for comparability with other assessment instruments) in the form of a statement (e.g., “Overall, I am very satisfied with my stay on the ward”), while items 4–25 are in the form of a wish or request, e.g., “The therapist should take me more seriously”).

The BEST for parents comprises 22 items and ratings are given on the same 5-point scale. Analogously to the adolescent version, items 1–3, and 22 are formulated as statements and items 4–21 as wishes. Because high values in the “request” items mean high dissatisfaction, these items were reverse coded for the statistical analyses. Thus, high values always mean high satisfaction in the remainder of this article.

Additionally, both questionnaires contain a stigma question (“afraid that others will find out about my stay/our child’s stay”) and items asking for complementary information, e.g., “agreed to admission”, “duration of stay was too short/exactly right/too long”.

The “Therapy Evaluation Questionnaire” (TEQ) [18] is available in a patient, a parent and a therapist version. The patient version comprises 20 items and the parent’s version 21 items. Three subscales are distinguished for the patient version labelled “treatment success”, “relationship with therapist”, and “general treatment conditions”. The subscales for the parent version are “treatment success” and “course of treatment”. Ratings are given on a 5-point scale ranging from 0 = “not at all/never” to 4 = “exactly/always”. Some items are negatively formulated and must be reverse coded for the analysis.

### Statistical analysis

To explore the dimensional structure of the two questionnaires we first assessed the fit of a one-dimensional model to test whether all items of an instrument measure the same latent variable. We then assessed an exploratory correlated factor model with two to five factors to test whether the items assess different correlated constructs. Model fit was evaluated based on goodness of fit-criteria using the comparative fit index (CFI), the Tucker–Lewis index (TLI), and the root mean square error of approximation (RMSEA). A CFI ≥ .95, an RMSEA value ≤ .06, and a TLI ≥ .95 are considered as indicating a good fit; a reasonable fit is indicated for values of CFI ≥ .90, and RMSEA ≤ .08 [41, 42]. The best-fitting model for each questionnaire was then selected and further analyzed as a bifactor model. Bifactor models assume that all items load on one general dimension, but that there are remaining sources of covariation because of common characteristics of certain item sets. Statistical indices to evaluate bifactor models, i.e. to separate and compare several sources of variance due to the general factor and to the specific factors alone, were coefficient omega, omega hierarchical and the concept of explained common variance (ECV). All coefficients were calculated according to the formulas given in Rodriguez et al. [43]. The factor analysis models were estimated with M*plus* 7.4 [44] using the weighted least square and mean and variance-adjusted (WLSMV) estimator where items are treated as ordered-categorical and a missing data estimation based on full-information-maximum-likelihood (FIML) is provided. Mean scores of the total scale or the subscales were computed by the mean of the available items for each scale (given that the required number of items for the total scale was available). All other statistical calculations were performed with the Statistical Analysis System (SAS) version 9.4.

## Results

### Descriptive statistics for items of the adolescent version (BEST-A)

The adolescents rated the overall treatment satisfaction (item 1) as being good (see Table 1 for a summary of all items). Highest satisfaction was in handling of confidential information and to be taken seriously by teachers, closely followed by taken seriously by therapist. The information about medication was also rated as quite satisfactory. On the lower end of the ratings, we found aspects of “hotel quality”, i.e. the quality of food and the decoration of the ward. Opportunities to be alone were less satisfactory. The sample size in item 5 (information about medication) suggested (indirectly) that 70% of the adolescents received medication. All items showed sufficient item-total correlations.

**Table 1:** Means, standard deviations (SD) and item-total correlations (r_it_) for the BEST items in adolescents

Items	N	Mean	SD	r_it_
1. Overall satisfied	1578	3.81	.97	.54
2. Stay on ward was helpful	1574	3.72	1.12	.46
3. Motivation to participate	1520	3.81	1.11	.32
4. Information about illness/problem	1563	3.17	1.34	.47
5. Information about medication	1102	3.71	1.32	.46
6. Taken seriously by therapist	1571	3.87	1.36	.54
7. Effectiveness of one-on-one sessions	1572	3.39	1.35	.60
8. Effectiveness of family sessions	1509	3.44	1.30	.54
9. Taken seriously by caregivers	1576	3.47	1.38	.60
10. Taken seriously by teachers	1197	4.02	1.25	.41
11. Handling of confidential information	1572	4.03	1.25	.51
12. Information about coercive measures	1522	3.34	1.28	.57
13. Leave regulations (weekdays)	1539	3.09	1.46	.54
14. Food quality	1577	2.39	1.38	.31
15. Sanitary facilities	1574	3.18	1.41	.49
16. Privacy when parents visit me	1507	3.22	1.43	.54
17. Decoration of the ward	1575	2.88	1.42	.50
18. School offer	1431	3.52	1.32	.47
19. Had a say in selection of therapies	1523	3.02	1.33	.55
20. Had a say in decoration of my room	1500	3.06	1.39	.53
21. Information about treatment after stay	1538	3.52	1.26	.57
22. Athmosphere among adolescents	1576	3.60	1.31	.39
23. Privacy respected	1575	3.26	1.39	.62
24. Opportunities to be alone	1579	2.83	1.40	.50
25. Goals of treatment discussed	1573	3.25	1.32	.64
26. Expectations fulfilled	1569	3.49	1.17	.43
27. Would come back	1558	3.54	1.44	.41

### Factor structure of the BEST-A items

Inspection of the goodness of fit indicators for the solutions with different numbers of factors (Table 2) revealed that the assumption of a single underlying factor was not tenable due to insufficient goodness of fit-values. The solution with two factors was close to acceptable fit, but the improvement for the 3-factor solution was substantial. Further increase in the number of factors showed even better fit according to the indicators, but improvement over the 3-factor solution was small and more importantly, the emerging additional factors were not convincing. Factor 4 in the 4-factor solution consisted of the item pair 23 und 24 (privacy). In the 5-factor solution, factor 5 again was constituted by the item pair 23 and 24, and factor 4 had a special focus on the therapist (items 6–8) which pointed to residual correlations between items but not to a distinct factor. Thus, the 3-factor solution was preferred and the factor loadings of this solution are displayed in Table 3.

**Table 2:** Goodness of fit-indices for factor analyses (FA) with the WLSMV estimator for adolescents (*n* = 1582)

Model	*Chi*^2^	*df*	CFI	TLI	RMSEA
Exploratory FA					
1 factor	6370.0	324	.792	.775	.109
2 factors	3360.2	298	.895	.876	.081
3 factors	1538.7	273	.956	.944	.054
4 factors	1267.9	249	.965	.951	.051
5 factors	1002.2	226	.973	.959	.047
Confirmatory FA^a^					
1 factor	6002.5	322	.805	.787	.106
2 correlated factors^b^	2706.3	321	.918	.910	.069
3 correlated factors	2123.9	319	.938	.932	.060
Bifactor model^c^	1302.8	297	.965	.959	.046

^a^All CFA models were estimated with two residual correlations, namely item pair 4 and 5, and item pair 23 and 24

^b^Factor 1 combined factors Therapeutic relationship and Environment; r_12_ = .55

^c^General factor and three specific factors

**Table 3:** Factor loadings of EFA with three factors: Adolescents

Items	F1	F2	F3
1. Overall satisfied		.29	**.71**
2. Stay on ward was helpful	.23		**.76**
3. Motivation to participate			.49
4. Information about illness/problem	**.54**		
5. Information about medication	.46	.23	
6. Taken seriously by therapist	**.73**		
7. Effectiveness of one-on-one sessions	**.81**		
8. Effectiveness of family sessions	**.67**		
9. Taken seriously by caregivers	.42	.32	
10. Taken seriously by teachers	.33	.33	
11. Handling of confidential information	.32	.40	
12. Information about coercive measures	.24	.46	
13. Leave regulations (weekdays)		**.55**	
14. Food quality		**.53**	
15. Sanitary facilities		**.72**	
16. Privacy when parents visit me		**.65**	
17. Decoration of the ward		**.71**	
18. School offer		**.53**	
19. Had a say in selection of therapies		**.53**	
20. Had a say in decoration of temporary room		**.70**	
21. Information about treatment after stay	.33	.43	
22. Atmosphere among adolescents		.44	
23. Privacy respected		**.71**	
24. Opportunities to have time alone		**.66**	
25. Goals of treatment discussed	.36	.42	
26. Expectations fulfilled			**.71**
27. Would come back			**.61**

Values > .50 in bold, values < .20 not shown

Factor 1 was labelled as “Therapeutic relationship” since it was determined mainly by items 4–8 referring to the therapist (relationship, giving information, therapy sessions) and (less pronounced) by the caregivers (item 9) who also play an important role in the treatment process. Items 10 (teacher in the clinic school) and 11 (handling of confidential information) showed about equal loadings on factors 1 and factor 2, but seemed to be connected to persons in the treatment process. The items 21 (information about treatment after stay) and 25 (goals of treatment discussed) also had indecisive loadings. By content, however, these items should be part of the “Therapeutic relationship” factor. Thus, factor 1 (and the subscale thereof) consists of items 4–11, 21, and 25 (10 items). Concerning factor 2, factor loadings were quite clear in constituting an environment/regulations factor consisting of the items 13–17, 20, 22–24 (9 items). In addition, item 19 (say in selection of therapies) had a substantial loading on factor 2 which was surprising since in our mind it should be related to the therapist factor but was seen also as belonging to “environment/regulations” by the adolescents. Due to this indecisive finding, this item was not attributed to a factor. Factor 3 was clearly structured with no relevant cross-loadings; the factor comprised items 1–3, 26, and 27 (five items) and was labelled “General satisfaction and treatment success”.

Three items were not part of a subscale: item 12 (information about coercive measures) that was attributed with a preference for factor 2, but the content does not seem to fit well; furthermore, item 18 (school offer) reflected a separate domain and should better be used as a single item; finally, item 19 for the reason given above. Hence, items 12, 18, and 19 were not part of a subscale, but of the total score. Inter-factor correlations were *r*_12_ = .56, *r*_13_ = .18, and *r*_23_ = .31. The mean value of the total score was 3.38 (*SD* = .73); concerning subscales, the mean values were 3.57 (*SD* = .88) for “Therapeutic relationship”, 3.06 (*SD* = .89) for “Environment”, and 3.67 (*SD* = .88) for “General satisfaction and treatment success”. The internal consistency of the total score (27 items) was Cronbachs α = .91; for the subscale “Therapeutic relationship”, α = .85, and for the two other subscales, α = .81 for each of them.

### Descriptive statistics for items of the parent version (BEST-P)

The parents were quite satisfied with overall aspects of the child’s stay (item 1, 22) (see Table 4 for a summary of all items). As for adolescents, the highest satisfaction was rated in handling of confidential information, but many items were also rated as good (and usually much better than the corresponding item in adolescents). On the lower end, the least satisfying item concerns the number of one-on-one sessions. Hence, the parents found their frequency too low and wanted more of them for their child. From the sample size in item 5 (information about medication) it can be concluded that 71.8% of their children received medication. All items showed good item-total correlations.

**Table 4:** Means, standard deviations (SD) and item-total correlations (r_it_) for the BEST items in parents

Items	N	mean	SD	r_it_
1. Overall satisfied	1989	4.21	.90	.53
2. Stay was helpful for child	1976	3.98	1.01	.47
3. Stay was helpful for us	1980	3.96	1.04	.49
4. Information about illness/problem	1977	3.56	1.35	.60
5. Information about medication	1434	3.95	1.29	.55
6. Taken seriously by therapist	1977	4.06	1.32	.69
7. Effectiveness of one-on-one sessions	1919	3.55	1.33	.73
8. Effectiveness of family sessions	1939	3.60	1.34	.73
9. Effectiveness of stay	1970	3.49	1.34	.74
10. Had a say in selection of therapies	1958	3.59	1.34	.72
11. Handling of confidential information	1965	4.35	1.10	.63
12. Information about coercive measures	1812	3.82	1.34	.65
13. Had a say in decoration of his/her room	1774	3.76	1.28	.61
14. Leave regulations (weekend)	1725	3.75	1.41	.53
15. Sanitary facilities	1794	3.83	1.30	.51
16. Decoration of the ward	1916	3.52	1.36	.48
17. Had a say in determining date of discharge	1964	3.71	1.40	.63
18. Number of one-on-one sessions (child)	1950	3.18	1.44	.61
19. Privacy of child respected	1919	3.95	1.16	.70
20. Opportunities for child to be alone	1953	3.82	1.24	.61
21. Goals of treatment discussed	1983	3.51	1.40	.74
22. Would bring child back	1982	4.29	1.11	.48

### Factor structure of the BEST-P

Analogously to the results in adolescents, the inspection of the goodness of fit indicators for the solutions with different numbers of factors (Table 5) revealed that the assumption of a single underlying factor was not tenable, the solution with two factors was close to acceptable fit, and the improvement for the 3-factor solution was substantial. Further increase in the number of factors showed even better fit according to the indicators but, again, improvement over the three-factor solution was small and the emerging additional factors seemed not substantial. In a 4-factor solution, factor 4 consisted of item pairs 4, 5, and 15, 16. In a 5-factor solution, the item pairs split into factor 4 constituted by items 4 and 5, and factor 5 by items 15 and 16. Thus, the 3-factor solution was retained and the two item pairs were included as residual correlations in further confirmatory factor analyses. Table 6 presents the item loadings onto the three factors.

**Table 5:** Goodness of fit-indices for factor analyses (FA) with the WLSMV estimator for parents (*n* = 1998)

Model	*Chi*^2^	*df*	CFI	TLI	RMSEA
Exploratory FA					
1 factor	9483.9	209	.845	.829	.149
2 factors	4225.5	188	.933	.917	.104
3 factors	2151.7	168	.967	.955	.077
4 factors	1549.3	149	.977	.964	.069
5 factors	1080.4	131	.984	.972	.060
Confirmatory FA^a^					
1 factor	7096.2	206	.885	.871	.129
2 correlated factors^b^	3461.8	204	.946	.939	.089
3 correlated factors	2115.6	202	.968	.964	.069
Bifactor model^c^	1372.3	183	.980	.975	.057

^a^All CFA models were estimated with three residual correlations, namely item pair 4 and 5, item pair 15 and 16, and item pair 19 and 20

^b^Factor 1 combined factors Therapeutic Relationship and Environment; r_12_ = .60

^c^General factor and three specific factors

**Table 6:** Factor loadings of EFA with three factors: Parents

Items	F1	F2	F3
1. Overall satisfied	**.82**		
2. Stay was helpful for child	**.90**		
3. Stay was helpful for us	**.87**		
4. Information about illness/problem		**.80**	
5. Information about medication		**.78**	
6. Taken seriously by therapist		**.84**	
7. Effectiveness of one-on-one sessions		**.91**	
8. Effectiveness of family sessions		**.93**	
9. Effectiveness of stay	.25	**.67**	
10. Had a say in selection of therapies		**.61**	.21
11. Handling of confidential information		**.69**	.31
12. Information about coercive measures		**.51**	.34
13. Had a say in decoration of his/her room		.34	**.53**
14. Leave regulations (weekend)		.34	.42
15. Sanitary facilities			**.76**
16. Decoration of the ward			**.75**
17. Say in determining date of discharge		**.51**	.25
18. Number of one-on-one sessions (child)		**.57**	
19. Privacy of child respected		.43	**.60**
20. Opportunities for child to be alone		.34	**.59**
21. Goals of treatment discussed		**.62**	
22. Would bring child back	**.65**		

Values > .50 in bold, values < .20 not shown

As in the adolescent solution, the factor “General satisfaction and treatment success” was clearly structured except a small cross loading in item 9; the factor comprised items 1–3, and 22 (four items). The second factor was named “Relationship with therapist” since the items 4–11 that were all related to the therapist have substantial loadings on this factor. Items 17, 18 and 21 were also connected with the therapist. Items 13–16 and items 19 and 20 mainly constituted the third factor. These items addressed the environment and regulations and the factor was labelled “Environment”. The item 12 (information about coercive measures) revealed a preference for factor 2, but due to the unclear loadings and analogously to the adolescent scale, this item was not attributed to a subscale, but part of the total score. The inter-factor correlations were *r*_12_ = .58, *r*_13_ = .22, and *r*_23_ = .49. The mean value of the total score was 3.79 (*SD* = .83); concerning subscales, the mean values were 3.67 (*SD* = 1.00) for “Relationship with therapist”, 3.78 (*SD* = .99) for “Environment” and 4.11 (*SD* = .85) for “General satisfaction and treatment success”. The internal consistency of the total score (22 items) was Cronbachs α = .94; for the subscale “Relationship to therapist”, α = .92, and for the two other subscales, α = .85 for each of them.

### Relationship of satisfaction scores with age and sex

Results of the correlational analysis between age of the adolescents and the subscale scores for adolescents as well as of parents revealed very low correlation coefficients and all of them were non-significant (Table 7). With regard to sex, the correlations (that were used instead of a t-test for a compact description of the results) were not significant as well, with the exception of the total score (*p* ≤ .05) and of the subscale environment (*p* ≤ .01) in adolescents. When converted into differences of mean scores, the girls rated their total satisfaction lower than the boys with a difference of .13; in the subscale environment, the difference is .17. Quantified as effect sizes (Cohens *d*), these differences were shortly below a low effect size (*d* = .18 and .20, respectively).

**Table 7:** Correlations between adolescent factors and parent factors and with age and sex

Total score/subscale (S)	Adolescents			Parents				Age	Sex
	S2	S3	Total	S1	S2	S3	Total		
Adolescents									
S1: Therapeutic relationship	.65	.42	.89	**.34**	.25	.22	.34	− .03	− .06
S2: Environment		.37	.87	.23	**.29**	.13	.27	− .01	− .10
S3: Gen. satisfaction, success			.61	.35	.26	**.36**	.38	.04	− .03
Total score				.36	.32	.26	**.39**	− .02	− .09
Parents									
S1: Relationship with therapist					.65	.49	.95	− .03	− .03
S2: Environment						.29	.81	− .03	− .04
S3: Gen. satisfaction, success							.61	− .05	− .03
Total score								− .04	− .04

Correlation coefficients between factors are based on *n* = 815; for age, *n* = 737; for sex, *n* = 747. All correlation coefficients between factors are significant at *p* < .0001; correlations with age are not significant (all *p* > .15); correlations with sex are not significant except for adolescents in the total score (*r* = − .09, *p* = .019) and in S2: Environment (*r* = − .10, *p* = .008)

### Convergent validity: correlations between BEST and TEQ scores

For the adolescent sample, the correlation between the total scores of BEST-A and TEQ-Patient was *r* = .59. Concerning subscale scores, there was a high correlation in the subscales “General satisfaction and treatment success” (BEST) and “Treatment success” (TEQ-Patient) with *r* = .79. The correlation between the two subscales assessing “Therapeutic relationship” was *r* = .48. The two roughly corresponding subscales “Environment” (BEST) and “Treatment conditions” (TEQ-Patient) revealed a moderate correlation with *r* = .39.

In the parent sample, the two total scores were highly correlated with *r* = .66 and the two subscales addressing treatment success revealed *r* = .71. The TEQ-Parent subscale “Course of treatment conditions” correlated at *r* = .51 with the BEST-P subscale “Environment” and at *r* = .69 with the BEST-P subscale “Relationship to therapist”. All correlation coefficients mentioned in this section were significant with *p* ≤ .001.

### Concordance between adolescent and parent perspectives

The correlation coefficients between satisfaction scores of adolescents and parents are displayed in Table 7. For the total score, the association between adolescents and parents was moderate with *r* = .39. Concerning subscales, the correlations between corresponding subscales were slightly lower (.34, .29, and .36) and the correlations between the subscales addressing the same content were always the highest.

Since these correlations between sum scores were subject to measurement error and were therefore attenuated, it was also attempted to model the parent and child perspectives as latent constructs within a structural equation modelling (SEM) approach. For estimating the relationship between the overall-satisfaction (corresponding to the total scores), a second-order factor model was used. The three factors found for the adolescents and the three factors found for the parents were defined by the respective items as assigned above. These first-order factors were indicators of the second-order factor representing the “total satisfaction”, separately for adolescents and for parents. The correlation between these two second-order factors provided the association on the latent level. The second approach simply looked at the correlation between the factors by estimating a joint model with the three factors defined as above for adolescents and for parents, but without the second-order structure.

The fit estimates of the second-order model were: Chi^2^(1114) = 2753.94, *p* < .0001, RMSEA = .043, CFI = .945, TLI = .942, indicating an acceptable to good fit. The estimated correlation between the two second-order factors was *r* = .48. Concerning the simple model with three correlated factors in each sample, the fit estimates were: Chi^2^(1106) = 2228.52, *p* < .0001, RMSEA = .035, CFI = .963, TLI = .960, indicating good fit. ‘Therapeutic relationship’ correlated at *r* = .40 with the respective view of their parents; for ‘environment’, the correlation was *r* = .36, and for the factor ‘general satisfaction and treatment success’, the correlation was *r* = .49. As in the case with the manifest sum scores, the correlations between the subscales addressing the same content were always the highest.

Taken together, however, these latent relationships that were not attenuated by measurement error provided no strong improvement over the correlation coefficients between the manifest sum scores, since increase in values was only around .10 and the latent correlations between the two perspectives still remained on a moderate level.

### Confirmatory factor analyses and bifactor models

Before the goodness of fit (and interpretability) of bifactor models was evaluated, a comparison assessment for the goodness of fit of the correlated factor models was conducted. The final model resulting from exploratory factor analyses (EFA) was the model with three correlated factors in both samples of adolescents and parents, with an item-factor composition as described above. In addition, some residual correlations were allowed in the model for item pairs with strong residual correlations suggested by modification indices in M*plus*, results from EFA, and conclusive interpretation from item content (see information on the item pairs in Table 2 for the adolescents and Table 5 for the parents).

The three correlated factors model revealed an acceptable fit in adolescents (Table 2) and an acceptable to good fit in parents (Table 5). The bifactor models with a general factor and three specific factors had a much better fit and the improvement against the model with three correlated factors was remarkable in the adolescent sample and substantial in the parent sample (see Tables 2 and 5). Factor loadings of the bifactor solution in adolescents are given in Table 8.

**Table 8:** Factor loadings of the confirmatory bifactor model (general factor G and three specific factors S1–S3, all factors uncorrelated): Adolescents

Items	G	S1	S2	S3
1. Satisfied overall	**.52**			**.64**
2. Stay on ward was helpful	.44			**.68**
3. Motivation to participate	.31			**.45**
4. Information about illness/problem	.48	.**31**		
5. Information about medication	**.50**	.17		
6. Taken seriously by therapist	**.57**	**.52**		
7. Effectiveness of one-on-one sessions	**.60**	**.65**		
8. Effectiveness of family sessions	**.56**	**.48**		
9. Taken seriously by caregivers	**.67**	.21		
10. Taken seriously by teachers	**.52**	.13		
11. Handling of confidential information	**.63**	.10		
12. Information about coercive measures	**.67**			
13. Leave regulations (weekdays)	**.60**		.19	
14. Food quality	.29		**.45**	
15. Sanitary facilities	.48		**.52**	
16. Privacy when parents visit me	**.59**		**.32**	
17. Decoration of the ward	**.50**		**.48**	
18. School offer	**.54**		.15	
19. Had a say in selection of therapies	**.66**			
20. Had a say in decoration of my room	**.58**		**.31**	
21. Information about treatment after stay	**.67**	.10		
22. Athmosphere among adolescents	.44		.17	
23. Privacy respected	**.68**		.19	
24. Opportunities to be alone	**.55**		.22	
25. Goals of treatment discussed	**.74**	.10		
26. Expectations fulfilled	.40			**.65**
27. Would come back	.40			**.57**

In bold are values > .50 on G and values > .30 on S1–S3; all loadings are significant at *p* ≤ .01

All items showed sufficient to strong loadings on the general factor, with many items having loadings > .50, and item 3 and 14 being at the lower end. The specific factor Therapeutic relationship was dominated by the items 6–8, but the remaining items of this factor still have significant loadings. Similarly, the specific factor environment showed significant loadings on all items of this factor, with a stronger weight on the “hotel quality” (items 14, 15, 17). The factor loadings on the third specific factor were pronounced and pointed towards the interpretation that these items were less absorbed by the general factor. Interestingly, some items that were not clearly attributable in the exploratory 3-factor solution, e.g. items 11, 12, 18, 19, 21, 25, exhibited strong loadings on the general factor; item 25 (goals of treatment discussed) even had the highest loading on the general factor and seemed to represent a core facet of the general construct satisfaction.

Concerning the parents, the factor loadings of the bifactor solution are given in Table 9.

**Table 9:** Factor loadings of the confirmatory bifactor model (general factor G and three specific factors S1–S3, all factors uncorrelated): Parents

Items	G	S1	S2	S3
1. Satisfied overall	**.54**	**.66**		
2. Stay was helpful for child	.45	**.76**		
3. Stay was helpful for us	.47	**.73**		
4. Information about illness/problem	**.64**		.29	
5. Information about medication	**.63**		.13	
6. Taken seriously by therapist	**.79**		.29	
7. Effectiveness of one-on-one sessions	**.76**		**.45**	
8. Effectiveness of family sessions	**.77**		**.47**	
9. Effectiveness of stay	**.74**	.26	**.35**	
10. Had a say in selection of therapies	**.81**		.05*	
11. Handling of confidential information	**.82**		− .02^n.s^	
12. Information about coercive measures	**.79**		− .16	
13. Had a say in decoration of his/her room	**.67**			**.42**
14. Leave regulations (weekend)	**.60**			**.32**
15. Sanitary facilities	**.54**			**.44**
16. Decoration of the ward	.49			**.44**
17. Say in determining date of discharge	**.75**		.00^n.s^	
18. Number of one-on-one sessions (child)	**.69**		.17	
19. Privacy of child respected	**.76**			**.37**
20. Opportunities for child to be alone	**.65**			**.38**
21. Goals of treatment discussed	**.83**		.09	
22. Would bring child back	**.56**	**.51**		

*n.s.* not significant

In bold are values > .50 on G and values > .30 on S1–S3; all loadings are significant at p ≤ .001 except where indicated: *p ≤ .05

As for the adolescent solution, all items showed strong loadings on the general factor, but the values were substantially higher indicating a more homogeneous construct of general satisfaction in parents. The factor loadings on the first specific factor were pronounced and indicated some independence from the general construct. The specific factor relationship to therapist was dominated by the items 7 and 8, i.e., the effectiveness of the therapy seemed essential; some of the remaining items of this factor still had significant loadings, while others (e.g., item 17) had no further loadings on this specific factor. In contrast, the specific factor environment showed substantial loadings on all items of this factor. In parallel with the adolescent solution, the item 21 corresponding in content to item 25 in adolescents had the (numerically) highest loading on the general factor and, again, seemed to represent a core aspect of the general construct satisfaction.

### Tests on multidimensionality

The partitioning of variance explained by the general and the specific factors revealed for the adolescent sample a coefficient omega (reflecting all sources of common variance) of .94. The omegaH (the reliability of the general factor alone) was .84 and the omegas for the specific factors were also high (.89 for S1, .86 for S2, and .85 for S3). They shrank considerably to values of .16, .21 and .56, respectively, when the general factor was partialled out (omegaHS). The relative strength of the general factor as evaluated by the explained common variance (ECV) was calculated as .67. Therefore, omegaH and ECV both suggested that the vast majority of variance was accounted for by the general factor; the subscales provided some additional information.

Concerning the parent ratings, coefficient omega (reflecting all sources of common variance) was .97. The omegaH (the reliability of the general factor alone) was .90 and the omegas for the specific factors were also high (.90 for S1, .95 for S2, and .88 for S3). They shrank to values of .57, .05 and .25, respectively, when the general factor was partialled out (omegaHS). The relative strength of the general factor as evaluated by the ECV was calculated as .74. Analogously to the results in adolescents, the vast majority of variance was accounted for by the general factor and the subscales provided some additional information, in particular the factor General satisfaction and treatment success in both versions.

## Discussion

This study investigated the psychometric properties of a questionnaire for assessing patient satisfaction of adolescents and of parents with in-patient psychiatric treatment in large samples of adolescents and parents. The main results concerning the psychometric properties can be summarized as follows: (a) all items and item categories showed good psychometric quality and good item-total correlations; (b) exploratory factor analyses revealed three factors that were similar in content for patients and for parents: Therapeutic relationship, Environment, and General satisfaction and treatment success; (c) the relation to sex and age of the child was very low; (d) the expected positive and statistically significant correlations between scores on the BEST and scores on the TEQ gave evidence for good convergent validity. Concerning agreement between adolescents’ and parents’ perspectives, the correlation between adolescent’ and parents’ sum scores were moderate and did not increase substantially when measurement error was statistically removed. With regard to the dimensionality of the BEST, advanced factor analysis modelling favoured a bifactor model in both samples, with a strong general factor accounting for most of the variance and the specific factors providing limited additional information.

Regarding the replicability of the psychometric results of the longer BEST version [37], the results with this revised version were in support of good comparability. Item endorsement rates and item-total correlations were in good agreement in both versions. The factor structure with the three factors could be reproduced in the parent version and they also emerged in the adolescent version. However, the two additional factors “schooling” and “perceived attention and information” that were found in Keller et al. [37] in adolescents could not be replicated. The explanation for the missing school factor is simply that there were only two school items left in the revised version; these two items show a moderate residual correlation, but do not constitute a factor. The missing factor “perceived attention and information” was identified with a different method (PCA based on pairwise correlations) which may have caused a non-replicable factor in the current advanced factor approach, in combination with the omission of some items in the current version of the BEST.

The three factors fit quite well with two of the universal components suggested by Biering [24]: “satisfaction with the environment and the organisation of the services”, and “satisfaction with the adolescent–caregiver relationship”. These two factors seem to also appear in other studies, e.g., [3], as outlined in the introduction. The third component suggested by Biering [24], “treatment outcome”, has some similarity with our “general satisfaction and treatment outcome”, but treatment outcome is only covered by 1–2 items in the BEST. In our view, treatment outcome should not be assessed within satisfaction questionnaires and with the same answering format as for the other satisfaction items, but by specific questionnaires for assessing symptom severity, such as SDQ or YSR/CBCL (see examples in [3, 7]). However, some outcome items in satisfaction instruments may be useful as “control items” (like our item 2 in adolescents), but the two constructs should be kept separate. Only in this case, studies on the relationship between treatment satisfaction and outcome seem valid, as well as further analyses of the type “which aspect of satisfaction predicts treatment outcome most” (unfortunately, no such outcome data were available in the present study, since the hospitals were not able to assess symptom severity at the same time). Nonetheless, studies on the relationship between treatment satisfaction and treatment outcome found only small to non-existent correlations, dependent on the used questionnaires and on self- vs. external ratings [3, 23, 24], although some studies report low to moderate correlations [7, 15].

Similarly, no relation was found between satisfaction scores and age of the adolescents. Differences according to sex were minimal and partly significant in adolescents, but below a small effect size. These results are consistent with findings in other studies [7, 9, 24, 45]. These studies also found the influence of diagnosis on differences in satisfaction to be of minor importance; however, most studies only used a rough grouping of internalizing and externalizing disorders, and the evaluation of specific diagnostic subgroups may yield different insights, e.g., the satisfaction with a family-based inpatient treatment for adolescent anorexia nervosa [46].

Concerning external validity, the study on the subsample that filled-in both questionnaires, the BEST and the TEQ, showed that scores on the BEST were adequately and in a differentiated way related to scores of the TEQ subscales, suggesting strong evidence for convergent validity. In addition, the values for correlation coefficients were generally similar to those in Keller et al. [37].

The correlation between child and parent perspectives was moderate, even after correcting for measurement error in a structural equation model. The correlation in a range between .30 and .40 agrees well with results from other studies [7, 18] and our previous results [37], although the findings in the research literature cover a wide range from a not-significant correlation [47] up to a (corrected) correlation of .80 [3]. Interestingly, this moderate range of agreement corresponds to the moderate correlations (with a wide range) that were found in the agreement of reporting symptom severity and functional impairment (c.f. [48]) or in the SDQ [49]. Qualitative analyses revealed some agreement, but also divergence regarding the criteria by which services are evaluated [50]. In conclusion, low agreement indicates that adolescents and parents have different needs and perceive satisfaction in different ways [3, 23, 24]. Consequently, the evaluation of only one informant is not sufficient to reveal a comprehensive evaluation of treatment satisfaction.

The second major aim addressed the dimensionality of the BEST. While a model with three correlated factors was adequate in fit in both samples, the assumption of a common underlying factor and three specific factors, as modelled with a bifactor approach, seemed superior. According to this model, the general factor accounted for the majority of variance and showed high reliability, while the reliability of the subscales turned out to be low after the general factor has been partialled out. However, the subscale General satisfaction remained substantial in reliability.

The clear difference between Therapeutic relationship and Environment on the one hand and General satisfaction on the other hand may not only be explained by a different perception of general vs. specific aspects of satisfaction, but also by the different wording of the items. The items of the first two factors were asked in form of a future request, and the items of the third factor are statements with regard to the current stay. Thus, an additional method component caused by the different wording seems apparent.

Since other studies on patient satisfaction did not use bifactor models and related statistical indices to quantify the “degree of unidimensionality”, our results cannot be compared directly. However, our strong general factor fits quite well with the conclusions of other studies on factor structure that found moderate to high correlated factors suggesting a unidimensional structure.

Some limitations need to be discussed. The composition of the sample and thus the representativeness of the sample must remain uncertain. It is largely unknown how many adolescents or parents have been asked to fill in the questionnaires in each hospital, and how many persons returned them. Feedback from the hospital staff indicated that the number of emergency stays and short stays in general might be underrepresented. Furthermore, there was a self-selection of hospitals taking part in the assessment. Thus, neither hospitals nor patients were picked at random. Nonetheless, the hospitals seem to be a selection of “usual” state hospitals with the typical clientele of patients for in-patient treatment. In addition, the usefulness of the satisfaction results for an internal evaluation is not so limited, since the heads and staff of hospitals are usually well informed about the number of admissions, the diagnostic composition and acuteness of the patients on the wards, as well as about further background variables including personal resources and structure quality. Thus, they rely on comprehensive experience, when they evaluate and compare satisfaction scores from, e.g., different wards within their hospital. For the same reason, it can be problematic to use satisfaction scores for benchmarking hospitals on a state or national level, without taking these background variables and the representability of the assessed cases into account. While the descriptive statistics could be biased by sample selection, the results concerning factorial structure of the BEST should be largely independent from them.

An important limitation is the lack of external validation data. Studies on the reliability and the factorial structure of satisfaction measures are ample, but there is a scarcity of studies when it comes to external validation of the individual responses by data on the hospital level. While therapeutic relationship is obviously difficult to assess by external raters, it may be helpful to explore differences in the “hotel quality”, e.g., by judging the environment by independent raters, or by comparing patients’ ratings before and after a major renovation of facilities took place. An older, but nice example from India in the 1970s is mentioned by Lebow [10] where a seemingly good and a bad hospital were compared and the better care was rated more positively. With regard to bifactor analysis, bifactor models were criticized for producing anomalous results under some circumstances [51] and for their tendency to show superior goodness of fit in model comparison studies [52]. However, our bifactor model is not intended to represent the structure of the construct satisfaction, but solely for reflecting psychometric properties, i.e., to inform about the degree to which the BEST yields a univocal total score and the extent to which the subscales yield reliable scores after accounting for the general factor.

Future research should try to also include short-term patients that may have been underrepresented in this study, along with additional variables such as type of mental disorder that may help in exploring further possible subgroup differences. The presentation of the BEST on a tablet-based solution that is currently created could facilitate the assessment procedure and therefore improve the number and quality of data. With more hospitals taking part it would be possible to perform multi-level analyses (as was done by Brown et al. [3], who based their multi-level analyses on 41 care providers), since patients are nested within hospitals and a hierarchical analysis gives further statistical power to detect effects.

Taken together, the BEST questionnaires seem reliable and are presumably valid instruments to assess adolescent and parent satisfaction with in-patient treatment. Furthermore, it not only addresses “classical” satisfaction issues such as therapeutic relationship and hotel quality, it also addresses today’s central topics such as children’s rights and treatment participation which has improved during recent years, but is still away from being common in in-patient treatment [53]. For practical application within the context of quality management (QM), the use on the item level often seems more appropriate. In our experience, quality managers prefer to compare mean values on single items and to plan specific interventions in order to overcome insufficiencies. If there are important differences in mean values in, for example, the ratings of “information about medication” between wards/hospitals, or ratings for “leave regulations on weekends” are unexpectedly low, the QM can address these difficulties by checking the procedures associated with these issues and act within a common strategy to improve them. Progress in these identified specific problem areas can also be controlled better after an adequate time period. Furthermore, concrete deficits, such as a comparatively low rated “decoration of the ward”, can be communicated better to the hospital administration than subscale scores. For scientific purposes, the use of subscale scores or of the total score seem preferable, since they are usually more reliable than single items. As outlined above, the usage of the total score seems superior because of the high explained variance by the general factor, but the subscale scores may provide additional information.

## Data Availability

The dataset analysed during the current study are available from the corresponding author on reasonable request.
